# Exploring the jasmonic acid signaling pathway: the role of *CsAOS* in modulating trichome density in cucumber

**DOI:** 10.1093/hr/uhag111

**Published:** 2026-03-30

**Authors:** Muhammad Ahmad, Li Shan, Sen Li, Yuming Dong, Yaru Wang, Menghang An, Lin Yang, Tiantian Pei, Yingqi Shi, Yibing Zhao, Hao Xue, Xinyue Ma, Huazhong Ren, Xingwang Liu

**Affiliations:** Department of Vegetable Science, College of Horticulture, China Agricultural University, Beijing 100193, China; Sanya Institute of China Agricultural University, Sanya 572000, China; Department of Vegetable Science, College of Horticulture, China Agricultural University, Beijing 100193, China; Department of Vegetable Science, College of Horticulture, China Agricultural University, Beijing 100193, China; Sanya Institute of China Agricultural University, Sanya 572000, China; Department of Vegetable Science, College of Horticulture, China Agricultural University, Beijing 100193, China; Department of Vegetable Science, College of Horticulture, China Agricultural University, Beijing 100193, China; Department of Vegetable Science, College of Horticulture, China Agricultural University, Beijing 100193, China; Department of Vegetable Science, College of Horticulture, China Agricultural University, Beijing 100193, China; Department of Vegetable Science, College of Horticulture, China Agricultural University, Beijing 100193, China; Department of Vegetable Science, College of Horticulture, China Agricultural University, Beijing 100193, China; Department of Vegetable Science, College of Horticulture, China Agricultural University, Beijing 100193, China; Department of Vegetable Science, College of Horticulture, China Agricultural University, Beijing 100193, China; Sanya Institute of China Agricultural University, Sanya 572000, China; Department of Vegetable Science, College of Horticulture, China Agricultural University, Beijing 100193, China; Sanya Institute of China Agricultural University, Sanya 572000, China

## Abstract

Jasmonic acid (JA) and its derivatives, including methyl jasmonate, are well-known plant growth regulators that mediate a wide range of physiological and developmental processes. Although the role of JA in regulating fruit trichome density has been recognized, the specific mechanisms underlying this remain to be fully understood. This study investigated the effects of various JA concentrations on trichome density at different developmental stages in cucumber (*Cucumis sativus* L.). Our findings revealed a dose-dependent increase in trichome density following exogenous JA application, with 1.5 mM JA showing the most significant effect at all stages. Conversely, the use of a JA biosynthesis inhibitor resulted in reduced trichome density, further highlighting the pivotal role of JA in trichome formation. Through transcriptomic analysis, we identified the *allene oxide synthase CsAOS* gene, which encodes an *allene oxide synthase*, as a key regulator of the JA biosynthesis enzyme preferentially expressed in trichomes. To investigate its functional role, we used CRISPR/Cas9-mediated knockout and overexpression strategies. Knockout of *CsAOS* in wild-type plants lead to a significant reduction in trichome density, whereas *CsAOS* overexpression in wild-type plants resulted in an enhanced trichome phenotype. These results provide novel insights into the molecular mechanisms governing trichome development in cucumbers, establishing *CsAOS* as a critical mediator of JA signaling in regulating trichome density. This study not only sheds light on the intricate relationship between JA and trichome development but also paves the way for future applications in plant breeding and genetic modification to improve pest resistance and herbivore defense.

## Introduction

Among Asian countries, many consumers highly value fruit trichomes of Cucurbitaceae species, which act as a determining factor for selecting cucumber (*Cucumis sativus* L.) cultivars in both commercial breeding programs and production centers. Trichomes are generally categorized as either glandular trichomes (GTs) or non-GTs, extend from the epidermal surface of plant organs, and differ in size, shape, chemical constituent, and species-specific localization [1–4]. Trichomes have various functions: they reflect light, they may reduce leaf wetness, they may secrete substances, and they may also defend plants against herbivores and pathogens [5, 6]. Jasmonates, such as jasmonic acid (JA) and its methyl ester derivatives (methyl jasmonate (MeJA)), are plant hormones regulating a variety of physiological and developmental responses. For instance, jasmonates stimulate storage organ formation, including tubers, and stimulate anthocyanin production [7], and they also increase abiotic and biotic stress tolerance. Foliar application of different chemical components, including silver nitrate and sodium thiosulfate, can change trichome density in cucumber fruits [8] and in Arabidopsis and tomato (*Solanum lycopersicum* L.) [9, 10]. Jasmonates positively regulate Arabidopsis trichome development and JA in trichome patterning by regulating *GL3*, a key transcription factor for wound-induced trichome formation [11]. In addition, treatment with different hormones, such as gibberellic acid (GA), indole-3-acetic acid (IAA), MeJA, and Eth, promoted trichome formation and trichome density increase in fruits compared to the control [12]. Despite its positive role in trichome development, JA has been shown to inhibit cell elongation and acts as an antagonist to both GA and IAA [13, 14]. Studies in the tomato mutant jasmonic acid insensitive1 (*jai1*), a mutant impaired in JA signaling, have also uncovered multiple JA-regulated defense phenotypes, including the unusual patterning of GTs. This implies that JA is necessary to mediate plant defense response through GTs [15]. JA has also been involved in plant responses to abiotic stress, especially drought [16–18], and is necessary for the biosynthesis of secondary metabolites that contribute to defense responses. In addition, JA interacts with a variety of transcription factors and other phytohormones to coordinate and regulate plant defense responses [19].

The first enzyme in the pathway leading to JA biosynthesis pathway is *allene oxide synthase (AOS).* These allene epoxides are unstable and catalyze cyclization to deliver the key precursors of JA, namely cyclopentenone acids [20]. As an important component of the octadecanoid pathway, *AOS* has a pivotal role in the regulation of the biosynthesis and accumulation of all JA-related compounds in plants to assist plant defense [21]. The biosynthesis of JA follows a well-defined pathway involving *defective anther dehiscence1 (DAD1)*, *lipoxygenase (LOX)*, *s allene oxide cyclase (AOC)*, *oxophytodienoate reductase 3 (OPR3)*, and *coronatine insensitive 1 (COI1)* [22, 23], an F-box protein in plants that acts as the central receptor for the hormone jasmonate. *MYC domain protein 2 (MYC2), MYC3,* and *MYC4* are JA-responsive transcription factors, and they promote leaf senescence-associated genes and chlorophyll degradation enzymes [24, 25]. Notably, overexpression of *GmAOS* gene in tobacco increased *AOS*, peroxidase (POD) and chymotrypsin inhibitor (CI) activities and significantly increased the density of trichomes on leaves of transgenic tobacco group compared with control plants [26]. In addition, *AOS* is essential and sufficient for JA production, which drives trichome development in Arabidopsis [11].

In summary, the exogenous application of varying concentrations of JA highlights its role in modulating trichome density in cucumber fruits, with increased JA concentrations correlating with higher trichome density. Additionally*, the allene oxide synthase* (*CsAOS*) gene, a key component of JA biosynthesis, was identified from the RNA-seq data as a critical player in this process. This study investigated the functional role of the *CsAOS* gene, and phenotypic observations of *CsAOS* knockout and overexpression lines demonstrated that *CsAOS* plays a role in cucumber trichome development via the JA pathway. These findings provide a foundation for future studies aimed at improving cucumber crop quality through the targeted manipulation of JA biosynthesis and trichome-related pathways.

## Results

### Trichome density and morphological variations in response to jasmonic acid treatment in the 6101–4 line

**Figure 1:**
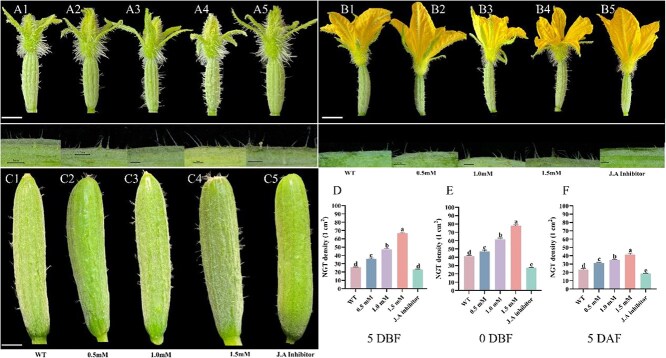
Cucumber ovary at different stages from the inbred line 6101–4 at three developmental stages. About 5 days before flower opening (A1–A5), flower opening day (B1–B5), and 5 days after flower opening (C1–C5) treated with varying concentrations of jasmonic acid (JA: 0.5, 1.0, and 1.5 mM) and a JA inhibitor. Scale bars represent 1 cm. Quantification of trichome density at the 5 DBF (D), 0 DBF (E), and 5 DAF (F) stages. Data are shown as means ± standard deviation (SD). Bars labeled with different letters indicate significant differences (*P* < 0.05) based on LSD test.

Significant responses to different JA concentrations were observed at different developmental stages of cucumber fruit in the inbred line 6101–4 (Fig. 1). We analyzed trichome formation at three developmental stages: 5 days before flower opening (5 DBF), flower opening (0 DBF), and 5 days after flower opening (5 DAF). This included the fruit at each stage with images of the effects of JA treatments (0.5, 1.0, and 1.5 mM) and treatment with the JA inhibitor.

The highest number of trichomes was obtained with JA treatment at 1.5 mM, which was significantly higher than that with the wild-type (WT) and other JA concentrations (5 DBF). The increased density of non-GTs, which were specifically evident on the fruit surface (Fig. 1 A1–A5), was directly related to fruit pubescence. Treatment with the JA inhibitor resulted in a reduction in trichome formation to almost the level observed in the untreated WT. As depicted in the data (Fig. 1D), the 1.5 mM JA treatment significantly increased the trichome density compared to the lower concentrations (0.5 and 1 mM) and the WT. Trichome numbers were lower in the JA inhibitor group but not significantly reduced (Fig. 1D).

In fruits with markedly increased trichome numbers, trichome formation was again most pronounced at 1.5 mM JA at the 0 DBF stage (Fig. 1 B1–B5). Trichome formation was intermediate with the 0.5 and 1.0 mM treatments, but was much reduced by the JA inhibitor, as with the WT. The highest trichome numbers occurred with the 1.5 mM JA treatment compared with the control and the lower concentrations (0.5 and 1.0 mM), as can be seen in Fig. 1E. Variation in the increase was shown by the least increase in the JA inhibitor treatment corresponding to this variation. In the later stage (5 DAF), the effect of JA on trichome formation continued, but the magnitude was less than in the earlier stages (Fig. 1 C1–C5), although even 1.5 mM JA still produced the greatest trichome density, but the increase was not as great as in the previous stages. Curiously, trichome formation at 1.5 mM was higher than that in the 0.5, 1.0 mM, and WT treatments, which were both lower and more variable in trichome numbers. As shown in (Fig. 1F), treatment with 1.5 mM JA resulted in the highest number of trichomes, which was statistically different from the lower JA concentrations and the JA inhibitor. The applied concentration of the JA inhibitor group also had the lowest trichome density, followed by the WT group.

In conclusion, application of JA clearly resulted in a concentration-dependent response in the inbred line 6101–4 at all three cucumber fruit developmental stages. In the highest JA treatment (1.5 mM), the concentration elicited a stronger increase in trichome density compared to the control and lower JA treatment (0.5 and 1.0 mM) at the 5 DBF. This effect persisted at the 0 DBF and 5 DAF stages. JA inhibitor treatment consistently decreased trichome formation. AT all stages, 1.5 mM JA was the most effective in enhancing trichome formation, with an overall robust dose dependence of JA in the 6101–4 line, and a significant decrease in trichome density was observed under JA inhibitor conditions.

### Trichome density and morphology modified in response to jasmonic acid treatment in the CCMC line

The effect of JA on trichome formation was also analyzed in Changchunmici (CCMC) at three different developmental stages of fruit, 5 DBF, 0 DBF, and 5 DAF. Trichome density was also determined after treatment with different concentrations of JA (0.5, 1.0, and 1.5 mM) and a JA inhibitor.

**Figure 2:**
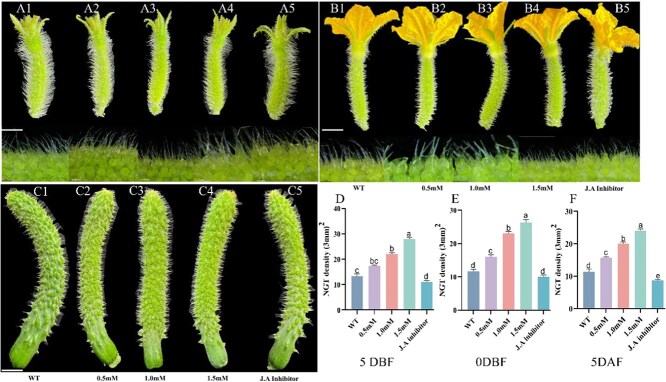
Cucumber ovary from the inbred line CCMC at three developmental stages. About 5 days before flower opening (A1–A5), flower opening day (B1–B5), and 5 days after flower opening and (C1–C5) treated with varying concentrations of jasmonic acid (JA: 0.5, 1.0, and 1.5 mM) and a JA inhibitor. Scale bars represent 1 cm. Quantification of trichome density at the 5 DBF (D), 0 DBF (E), and 5 DAF (F). Data are shown as means ± standard deviation (SD). Bars labeled with different letters indicate significant differences (*P* < 0.05) based on LSD test.

At 5 DBF, 1.5 mM JA presented with a greater increase in trichome formation of fruits at this early developmental stage, especially that of non-GT density relative to the WT and lower JA concentration (0.5 and 1.0 mM) (Fig. 2 A1–A5). The 0.5, 1.0, and 1.5 mM JA treatments gave moderate increases in trichome number, with the 1.5 mM dose showing a greater increase. Furthermore, JA inhibitor application resulted in a decrease in trichome formation compared to the WT, suggesting an inhibitory role of JA in trichome development.

The 1.5 mM JA treatment resulted in the greatest non-GT density at 0 DBF among all treatments (Fig. 2 B1–B5). In addition, the trichomes in the 1.0 mM JA group were significantly greater than those in the untreated WT and JA inhibitor groups. At a JA concentration of 0.5 mM, the effect was moderate and much less effective than that at higher concentrations in stimulating trichome growth. Again, the JA inhibitor treatment reduced trichome formation, as with its reduction in JA-mediated effects. The most significant enhancement in trichome density was observed at a concentration of 1.5 mM JA, which exhibited the highest number of trichomes across all treatments as well as under all the studied fruit stages (Fig. 2D–F). The lower concentrations of 0.5 and 1.0 mM JA showed intermediate effects on trichome density. The JA inhibition group showed a marked reduction in trichome density as compared to WT (Fig. 2D and F).

The response to JA was still evident at 5 DAF, but the effect of JA treatment was less pronounced. Although the 1.5 mM treatment still showed the highest trichome density, the difference between the 1.5 and 1.0 mM concentrations decreased (Fig. 2 C1–C5). However, contrary to effect attenuation, trichome count was maintained higher under 1.5 mM JA compared to any other treatment. The 0.5 mM JA treatment was moderate and, like the untreated control, the JA inhibitor treatment was drastically worse with significantly fewer trichomes.

A clear, concentration-dependent response to JA was observed in the CCMC inbred line for trichome formation with the 1.5 mM JA treatment, resulting in the highest trichome density for all stages. Especially at the 5 DBF and 0 DBF stages, 1.5 mM JA was very effective and rather successfully enhanced the trichome formation compared to lower concentrations (0.5 and 1.0 mM). These results confirmed that JA plays a role in trichome development and suppresses trichome formation with the JA inhibitor. The JA-induced response remained at 5 DAF but was less robust, indicating a stage-dependent response. Taken together, these results highlight the importance of JA in stimulating trichome formation in CCMC, with a clear dose-dependent response, especially during the early stages of fruit development.

### Scanning electron microscopy analysis in response to jasmonic acid treatment

**Figure 3:**
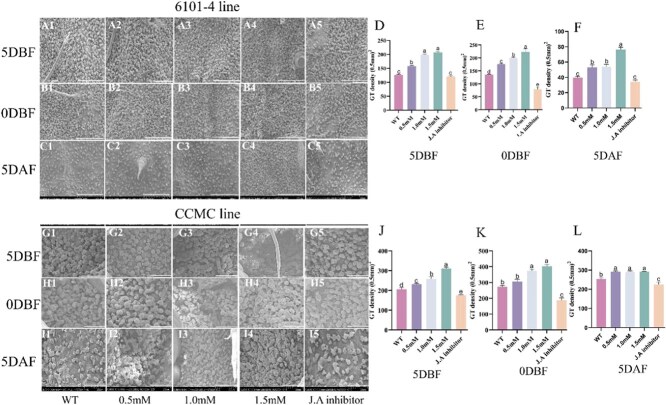
Scanning electron microscopy (SEM) analysis in 6101–4 and CCMC. (A1–A5), (B1–B5), and (C1–C5) images showing GTs on cucumber fruit surfaces from the 6101–4 line at 5 DBF, 0 DBF, and 5 DAF stages, respectively. Fruits were treated with varying concentrations of jasmonic acid (JA: 0.5, 1.0, 1.5 mM) and a JA inhibitor. The scale bar represents 500 μm. (G1–G5), (H1–H5), and (I1–I5) SEM images showing GTs on cucumber fruit surfaces from the CCMC line at 5 DBF, 0 DBF, and 5 DAF stages, respectively. The scale bar represents 200 μm. Quantification of GT density in the 6101–4 line, measured as the number of trichomes per 0.5 mm^2^ at the 5 DBF (D), 0 DBF (E), and 5 DAF (F) stages. Data are presented as mean ± standard deviation (SD), with bars labeled with different letters indicating significant differences (*P* < 0.05) based on the LSD test. Quantification of GT density in the CCMC line at the 5 DBF (J), 0 DBF (K), and 5 DAF (L). Data are shown as mean ± standard deviation (SD), with statistical differences indicated by letters (*P* < 0.05).

GT analysis was also conducted after JA treatment by scanning electron microscopy (SEM). For a detailed study of glandular trichome density, SEM images of two cucumber inbred lines (6101-4 and CCMC) treated with different JA concentrations (0.5, 1.0, and 1.5 mM) and JA inhibitors were obtained at 5 DBF, 0 DBF, and 5 DAF. The SEM images A1–A5, B1–B5, and C1–C5 representing the 6101–4 line and G1–G5, H1–H5, and I1–I5 representing the CCMC line clearly depict the alteration in the trichome density in these treatments (Fig. 3). SEM images at 5 DBF showed that the density of approximately 250 GTs per 3 mm^2^ in the 1.0 and 1.5 mM JA treatments, which was considered the highest density of GTs, especially in the 6101–4 line (Fig. 3D). At 0 DBF and 5 DAF, this effect was also observed, albeit more faintly at a later stage (Fig. 3E and F). The SEM images of the JA inhibitor treatment showed a substantial decrease in GT density relative to the WT, especially at 5 DBF and 0 DBF, which further corroborates the inhibition of JA signaling on trichome formation. The SEM analysis performed in the CCMC line showed a similar trend, with a maximum GT density of 400 per mm^2^ with 1.5 mM JA at 5 DBF (Fig. 3J). The SEM images for 0 DBF (Fig. 3K), however, also showed the same pattern, and 1.5 mM JA resulted in the greatest increase in trichome density. However, the difference between JA treatments decreased at 5 DAF (Fig. 3L). As with WT plants, the CCMC-invariant treatment reduced GT density specifically at later stages, similar to the WT treatment in CCMC.

### Increased expression of the *CsAOS* gene following JA treatment

**Figure 4:**
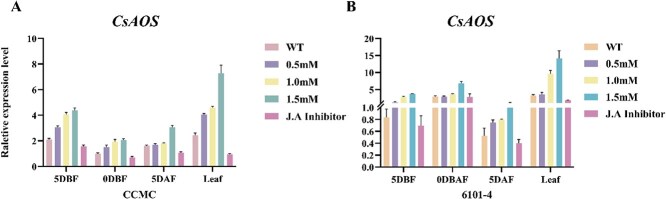
*CsAOS* expression in response to: (A) *CsAOS* response at of different JA concentrations in CCMC line. (B) in 6101–4 line at 5 DBF, 0 DBF, and 5 DAF of cucumber ovary and leaf.

Relative expression of the *CsAOS* gene in two cucumber lines, CCMC and 6101–4, was determined by varying concentrations of JA and JA inhibitor with tissue samples taken at different time points from cucumber (5 DBF, 0 DBF, and 5 DAF) and young leaves. The increase in *CsAOS* expression in the CCMC line is clearly dose-dependent with increasing JA concentrations (0.5, 1.0, and 1.5 mM) at most time points (Fig. 4A). *CsAOS* at 1.0 and 1.5 mM had the highest expression levels, especially in the leaf samples and at 5 DBF. Conversely, JA inhibitor treatment reduced *CsAOS* expression at all time points compared to the WT and JA treatments. However, 5 DBF and leaf samples were the most highly expressed, and the inhibitor decreased the expression in the WT. The trend of the JA concentration was similar, but with less variation between the JA concentrations in the 6101–4 line (Fig. 4B). Besides, 1.0 and 1.5 mM JA produced the highest *CsAOS* expression in most time points. The expression was reduced in the inhibitor condition compared to the WT; however, the leaf samples showed the highest expression of all the samples across all treatments. In general, treatment with JA led to a significant increase in *CsAOS* expression in both cucumber lines, with the largest *CsAOS* expression levels at 1.0 and 1.5 mM JA. Consistent with the involvement of JA in the regulation of *CsAOS* in these cucumber lines, JA inhibition suppressed gene expression. Finally, the results show that JA upregulates the *CsAOS* gene expression in a dose-dependent manner in both CCMC and 6101–4 cucumber lines, with the highest expression at 1.0 and 1.5 mM. This conclusion was confirmed using a JA inhibitor that effectively suppressed *CsAOS* expression. These results indicate that JA plays a key role in the regulation *CsAOS* expression in different cucumber lines and tissues.

### Cloning and sequence analysis of *CsAOS*

**Figure 5:**
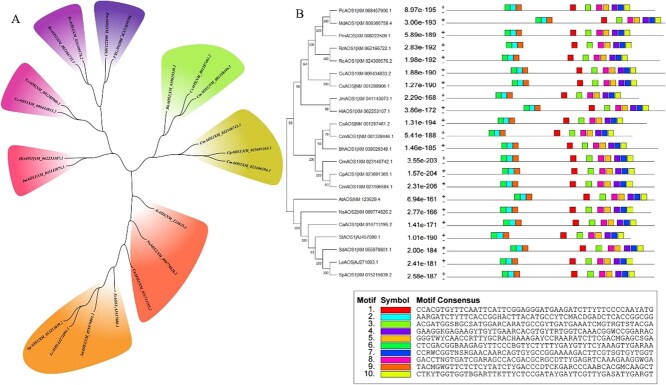
Evolutionary tree analysis (A), conserved and diverged sequence motifs (B) among species of *AOS.*

A phylogenetic tree analysis using cucumber *CsAOS* gene and selected *AOS* gene from other species indicates the diverse genetic relationship as shown in Fig. 5. The results indicated that the sequence similarity of *CsAOS* divided into clusters, which showed that the presence of the gene in other plant species (Fig. 5A). The analysis showed clustering of species based on both sequence homology and motif features. A detailed comparison of the motifs in the *CsAOS* gene with some of the orthologs from species such as *Solanum dulcamara*, *Lycopersicon esculentum*, *Malus domestica*, and *Cucumis melo* was performed (Fig. 5B). Additionally, the motif analysis demonstrates extensive sequence conservation and divergence among species. Most of these orthologs showed a high degree of conservation in the key motifs, particularly in motifs 1 and 2. Species within the same genera, such as *Cucumis* and *Solanum*, generally had highly similar motif compositions. However, orthologs from *Citrus* and *Rosa* showed more divergent motifs, which could suggest evolutionary adaptations in their respective biosynthetic pathways. While motifs 1 and 2 were conserved across species, some orthologs of *Capsicum annuum* and *Cucurbita moschata* exhibited variations in motifs 5 and 7, potentially indicating species-specific adaptations. These differences in motif distribution may be reflective of unique functional or regulatory aspects of *AOS* in these species. Motif analysis detects transcription factor binding sites in gene sequences and visualizes them as a phylogenetic tree, where *P*-values and the location of motifs are indicated. These motifs are shown in the form of colored bars that represent their positions and strands of DNA. The consensus sequence of all motifs is shown in Fig. 5B. A list of different species with scientific names used for phylogenetic analysis of *AOS* is listed in Supplementary Table S4.

### *CsAOS* orchestrates jasmonic acid biosynthesis by modification in the expression level

**Figure 6:**
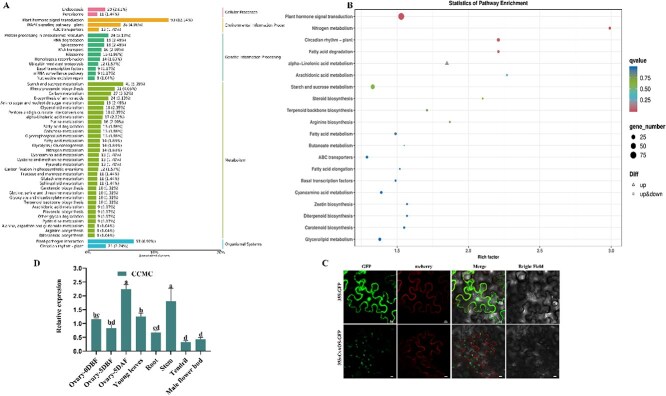
KEGG classification, subcellular localization, and expression pattern. (A) KEGG classification, (B) KEGG pathway, (C) subcellular localization of *CsAOS*-GFP in *N. benthamiana* leaf cells after 60 h (C, bar = 100 μm). (D) The relative transcript level of *CsAOS* in various cucumber tissues. O-0 DBF, ovary at 0 days flower; O-5 DBF, ovary at 5 days before flower; O-5 DAF, ovary at 5 days after flower; YL, young leaf; R, root; S, stem; T, tendril; MFB, male flower bud. Error bars represent SD from three biological replicates, ^*^P < 0.05.

The *CsAOS* gene was selected based on its expression profile from RNA sequencing (RNA-seq) analysis, and its functional role was further explored through transgenic analysis. Through transcriptome profiling, we focused on the *CsAOS* gene to further reveal the gene regulatory networks during leaf trichome development in cucumber WT, knockout, and *CsAOS* overexpression lines. Kyoto Encyclopedia of Genes and Genomes (KEGG) pathway analysis showed that the plant hormone signal transduction and alpha-linolenic acid metabolism pathways were significantly enriched (Fig. 6A), as well as the Mitogen-Activated Protein Kinase (MAPK) signaling pathway. All detected genes were upregulated giving a total of 17 genes related to the linolenic acid pathway. Most highly enriched pathways are mentioned in the Supplementary Table S2. To validate the differential expression patterns identified in the RNA-seq data, we selected key genes involved in the JA biosynthesis pathway. Quantitative real-time polymerase chain reaction (RT-qPCR) was performed using specific primers (The primers used for qRT-PCR are listed in Supplementary Table S3) designed for these genes to confirm their expression levels. The RT-qPCR results were consistent with the RNA-seq data, further supporting the accuracy and reliability of our findings (Supplementary Fig. S1). The *CsAOS* gene, selected from this pathway, underwent further functional analysis concerning leaf trichomes through both overexpression and knockout studies. Additionally, molecular functions also showed that significant enrichment was notably observed in starch and sucrose metabolism, circadian rhythm, nitrogen metabolism, and iron ion binding within the molecular functions (Fig. 6B).

### Subcellular localization of *CsAOS*

The complete coding region of *CsAOS* was fused with the coding sequence (CDS) of the Green Fluorescent Protein GFP reporter (*CsAOS*-GFP) and placed under the control of the 35S promoter (35S: *CsAOS*-GFP) to investigate the subcellular localization of *CsAOS*. In comparison, the control line expressing 35S:GFP showed a signal distributed throughout the entire cell (Fig. 6C). However, the expression of the *CsAOS*-GFP fusion protein produced a distinct chloroplast-specific signal in tobacco plants.

### *CsAOS* expression pattern

We investigated *CsAOS* function by quantifying its expression levels of *CsAOS* in cucumber roots, stems, young leaves, fruits, female and male flower buds, tendrils, and the cucumber line CCMC using quantitative reverse transcription PCR (qRT-PCR). *CsAOS* expression was detected in all the organs examined; however, the highest levels were observed in the fruit peel at 5 DAF, stem, and young leaves (Fig. 6D). However, the expression in these tissues was notably higher than that in the other organs that were sampled. In addition, transcript levels were analyzed at different stages of cucumber fruit development 5 DBF, 0 DBF, and 5 DAF. This showed that *CsAOS* was expressed at much higher levels at 5 DAF than at the earlier developmental stages (Fig. 6D). These results indicate that *CsAOS* may drive epidermal cell differentiation during leaf spine development. This suggests that *CsAOS* may play a role in cucumber trichome development.

### *CsAOS* positively regulates cucumber leaf trichomes

**Figure 7:**
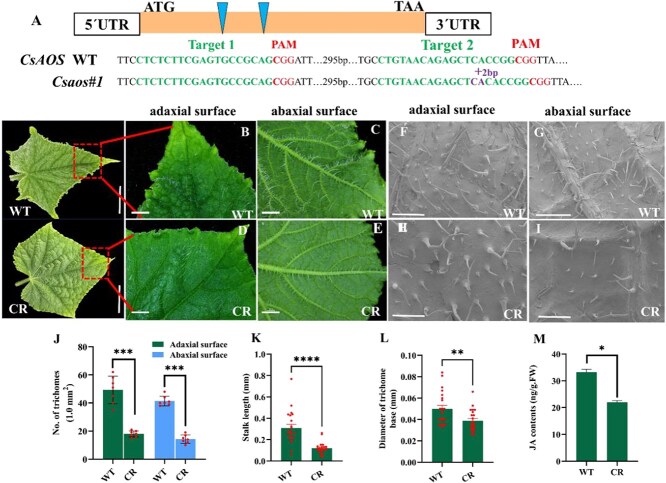
*CsAOS* alters trichome morphology. (A) Genetic modification within the *CsAOS* knockout (*aos*) line. There is one type of genetic modifications in the *aos* line that involves 2 bp addition from target (highlighted in purple). In addition, the base sequence marked in red is the PAM region. (B–E) Phenotypes of cucumber leaf in *CsAOS* knockout (CR) line in comparison with WT. (F–I) Scanning electron microscope observation of leaf trichomes in the *CsAOS* knockout (CR) line, in comparison with their WT (b–i bar = 1 cm). (J) Number of trichomes; *n* = 7. (K) Length of stalk. (L) Trichomes’ base diameter; *n* = 22. (M) JA contents analysis.

We used the CRISPR/Cas9 gene editing technology to generate the *CsAOS* knockout line, *aos*, to determine the biological function of *CsAOS*. All types of mutations in this led to the frameshift and premature termination of *CsAOS* translation (Fig. 7A). Specifically, on *aos* line leaves, adaxial and abaxial surface density of leaf trichomes was significantly lower compared to the CCMC WT (Fig. 7B–E), and the relevant results are shown in (Fig. 7J). This phenotypic change is further confirmed by SEM images showing the visually evident reduced number of trichomes in the *aos* plants, corroborating the functional disruption of *CsAOS* by the CRISPR technology. Subsequently, we used SEM and quantitative statistics to determine the impact of *CsAOS* knockout on the average diameter of the leaf trichomes’ base and stalk length (Fig. 7F–I). The mean diameter of the leaf trichomes’ base was decreased by significantly when *CsAOS* was knocked out relative to CCMC WT plants (Fig. 7L). The results suggest that *CsAOS* positively contributes to the morphogenesis of cucumber leaf trichomes, as well as to the reduction in stalk length (Fig. 7K).

### Overexpression of *CsAOS* increases cucumber trichome density

**Figure 8:**
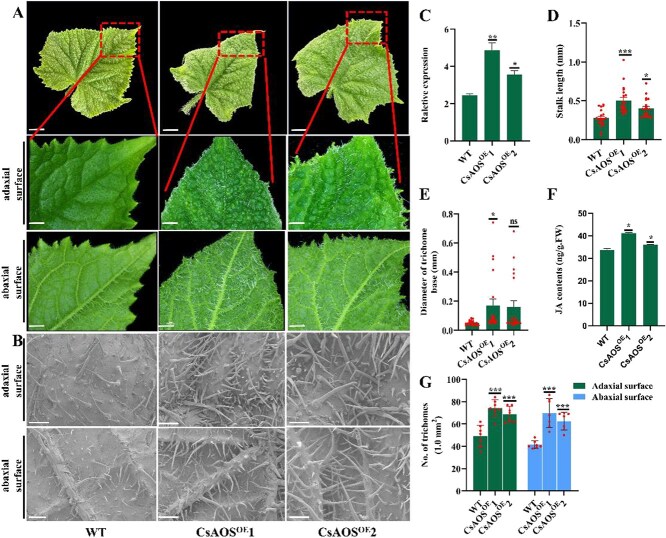
Critical role of overexpression of *CsAOS* in the morphogenesis of cucumber leaf trichomes. (A) Phenotypes of cucumber leaf trichomes within *CsAOS* overexpression (*CsAOS^OE^1* and *CsAOS^OE^2*) lines. (B) Scanning electron microscope observation of leaf trichomes within *CsAOS* overexpression (*CsAOS^OE^1* and *CsAOS^OE^2*), in comparison with their respective WT (A, b bar = 1 cm). (C) The relative transcript level of *CsAOS* within overexpression (Cs *CsAOS^OE^*) lines. Significance compared to WT was determined by Student’s t test; ^*^P < 0.05. (D) Length of stalk. (E) Diameter of trichomes’ base (*n* = 20). (F) JA content level. (G) Number of trichomes at adaxial and abaxial surface of leaves (*n* = 7).

To investigate whether the *CsAOS* gene could function in cucumber trichome morphology and density, Agrobacterium-mediated cotyledon transformation was used to introduce the 35S: *CsAOS* overexpression vector into the cucumber inbred CCMC line. To confirm successful integration of the transgene, the transgenic plants were selected using antibiotic resistance and genomically confirmed by PCR [27, 28]. By using this approach, we produced transgenic cucumber plants that overexpressed *CsAOS* so that we could further investigate its functional role in some of the developmental processes in the trichome development. Three positive overexpression lines were obtained. Detailed studies were conducted using two representative overexpression lines, *CsAOS^OE^1* and *CsAOS^OE^2*, whose *CsAOS* levels were much higher than in the WT line (Fig. 8C). In these two lines, a significantly higher number of trichomes were observed on both the adaxial and abaxial surfaces of the leaves compared to the WT plants (Fig. 8A). The WT plants exhibited a typical leaf structure with moderate trichome density on both sides. In contrast, the two overexpressing lines showed a marked increase in trichome numbers on leaf surfaces, indicating that *CsAOS* overexpression promotes trichome formation more than in the WT (Fig. 8A). Particularly, the *CsAOS^OE^1* plants showed increased trichome density on both leaf surfaces, as indicated in (Fig. 8G). SEM images, highlighted the increased number of trichomes on the leaves of *CsAOS^OE^1* and *CsAOS^OE^2* as compared to WT (Fig. 8B). These images reinforce the phenotypic observations and confirm that the overexpression of *CsAOS* leads to an increase in trichome density. The relative expression levels of *CsAOS* in WT and overexpression lines exhibited significantly higher *CsAOS* relative expression compared to the WT plants, with *CsAOS^OE^1* showing the highest expression level (Fig. 8C). Further, obvious differences were observed between the overexpression lines and WT plants in terms of stalk length in both overexpressed plants (Fig. 8D). A little difference was seen in trichomes’ diameter in the *CsAOS^OE^1* line but not in *CsAOS^OE^2* (Fig. 8E). The results confirm that the overexpression of *CsAOS* enhances trichome formation and change morphology to some extent and suggest that *CsAOS* plays a crucial role in regulating trichome density in cucumber leaf trichomes.

### Jasmonic acid content analysis measurements

Endogenous JA levels were subsequently measured in the *CsAOS* knockout transgenic lines (Fig. 7M) and in both *CsAOS* overexpression lines (Fig. 8F). The overexpression lines showed higher JA concentrations, whereas the knockout lines exhibited lower levels of JA. These results suggest that the overexpression of *CsAOS* induces transcriptional changes and indicates that *CsAOS* likely plays a role in leaf trichome morphogenesis through the JA-mediated pathway in cucumber.

## Discussion

### Jasmonic acid as a modulator of trichome development

Trichome development is driven by JA, a plant hormone integral to stress responses, especially during defense against herbivores. The increase in non-GTs and GTs observed across both 6101–4 and CCMC lines is in line with previous studies showing that JA regulates trichome density in a range of plant species (Figs 1–3). Plants require trichomes for defense, which provide a physical barrier against herbivores and improve transpiration, which is the primary means of water loss [29]. Therefore, JA may function as an important regulator of these protective structures during both cucumber leaf and fruit development. Furthermore, the results of this study also show that JA’s effect on trichome formation is dose-dependent. Concentrations of JA (1.5 mM) resulted in a greater induction of trichome formation than other experimentally studied concentrations, which supports the idea that the JA signaling pathway is also controlled by concentration thresholds to regulate trichome development. These findings are consistent with previous research in cucumber and other species, where the concentration of JA has been demonstrated to regulate trichome density in a concentration-dependent manner [12, 30, 31].

It has also been reported that other plant hormones such as GA, MeJA, and IAA influence trichome development [12, 32–34]. These results are also consistent with previous research showing that the *AaGL3-like* transcription factor has previously promoted trichome density by increasing the expression of trichome developmental genes *AaHD1* and *AaGSW2* in *Artemisia annua* [35, 36]. Likewise a previous study [37] demonstrated how *AaSEP1* concatenates JA and light signaling to trigger GST initiation. Trichomes are major defensive structures that prevent physical blocking of herbivores and decrease transpiration [30]. The JA treatment in this study enhanced both non-GTs and GTs, as observed in other species for which the induction of cell wall thickening, such as trichomes in other species, in response to MeJA has been shown [10, 31]. This indicates that JA is important for mediating the defense response of plants to cucumber fruit and leaf development.

The most interesting result was the stage-dependent variation in trichome formation, particularly the most pronounced increase at the 5 DBF stage. This coincides with the findings of previous studies showing that trichome density can be developmentally regulated in response to JA [38]. This effect gradually loses its importance at the 0 DBF and 5 DAF stages, implying a dynamic hormonal regulation of JA sensitivity during fruit maturation. In other species, such as *S. lycopersicum* (tomato), high trichome density was observed at early developmental stages, and a fruit-matured JA decreasing effect was also noted as well [10]. Other growth processes likely become more important in this stage-dependent attenuation, which apparently depends on shifts in the plant’s developmental priorities.

In addition, the observed increase in trichome density at higher JA concentrations (1.5 mM) coincides with earlier reports of trichome formation having a dose-dependent effect on JA treatment. For example, in *Cannabis sativa*, GT number increases due to higher concentrations of MeJA [31]. Similar to other species, studies on *A. annua* [35] and *Nicotiana benthamiana* [39] have also shown that JA signaling significantly modulates trichome density, and higher concentrations tend to result in higher trichome density. Our study also demonstrates the complexity of JA signaling, with time and developmental modulation of hormone sensitivity, as decreased trichome formation at later stages of fruit development was observed in this study. Research on other species, such as *A. annua* [37], also reported that the effect of JA is specifically stage-dependent, declining in a stage-specific manner. Perturbation of the shifting balance of hormone signaling during fruit maturation may explain the attenuation of trichome formation in this plant, which prioritizes other physiological processes.

The concentration- and stage-dependent effects, in addition to a possible basal molecular mechanism of JA-regulated trichome formation, likely involve complex transcriptional regulation. It has been reported that in *N. benthamiana*, *NbJAZ3* regulates *NbWo* and *NbCycB2* [39] downstream genes to negatively regulate GT development, indicating that trichome formation is precisely controlled through the regulation of trichome formation-related genes. This supports further investigation into transcription factors during cucumber JA-mediated trichome development, which shares some similarities with those in tomato [40]. The transcription factor *SlMYC1* has also been shown to function in both JA-mediated trichome formation and cell division in tomato [41]. In addition, DIECA (a JA biosynthesis inhibitor) showed that JA is essential for trichome formation. This study also showed that the inhibition of JA biosynthesis results in a reduction in trichome density [42]. This finding underscores the need for JA signaling to activate important genes such as *GLABRA3* (*GL3*) and *MYB* proteins that drive trichome initiation and maintenance [11, 43].

However, it has been demonstrated that the application of JA can increase trichome formation and density in many plant species. The concentration-dependent effect of JA on trichome formation is such that a higher concentration results in a greater increase in trichome density. JA-mediated trichome formation is regulated by a complex network of JA-responsive transcriptional networks, in which both positive and negative regulators are functional. JA application results in a trade-off in plant growth, but increases defense-related metabolites and resistance to herbivores and pathogens, making it a valuable tool in agriculture and biotechnology.

### *CsAOS* is involved in regulation leaf trichome development

Trichomes are non-branched and multicellular and, are found in different parts of plants, such as leaves, flowers, and fruits [44–46]. The expression patterns of *CsAOS* were investigated in this study (Fig. 6D). *CsAOS* was highly expressed in the stem, young leaf, and fruit peel, especially at 5 DAF. This expression pattern was similar to that of *VvAOS* in grapevines. *VvAOS* was expressed at higher levels in the pulp tissues of grapevines [47]. As *CsAOS* is a well-known early regulator of members of the cytochrome P450 family of proteins that are important for plant growth and defense [11, 48], we hypothesized that *CsAOS* also controls cucumber leaf and fruit epidermal cell growth and development.

*CsAOS* was found to be a key regulator of JA biosynthesis. KEGG pathway analysis showed that linolenic acid metabolism and plant hormone signaling were among the upregulated pathways, and consistent with this, they appeared to be involved in these processes. In addition, enriched genes involved in starch and sucrose metabolism and MAPK signaling imply that JA affect both metabolic processes and stress responses, and further investigation is required to determine the full role of JA. This finding highlights the broad role of *CsAOS* in JA biosynthesis, growth, and adaptation to stress.

We further confirmed the functional role of *CsAOS* in the regulation of leaf trichome development by creating a *CsAOS* knockout line via CRISPR/Cas9 gene editing (Fig. 7A). Our results demonstrated a marked reduction in both trichome density and morphological characteristics (e.g. spine diameter and stalk length) in *CsAOS* knockout lines, suggesting that *CsAOS* plays a crucial role in trichome initiation and morphogenesis (Fig. 7B–E and F–I). This finding is in line with previous research demonstrating that *AOS* is indispensable for JA biosynthesis and JA-driven trichome formation. Notably, *AOS* is the second enzyme downstream of the JA biosynthesis pathway, converting 13(S)-hydroperoxyoctadecatrienoic acid (13-HPOT) to unstable allene oxide, which is subsequently converted to 12-oxo-phytodienoic acid (OPDA) by *AOC* [49, 50]. Loss of *AOS* genes in *Marchantia polymorpha* causes reduced OPDA accumulation and decreased resistance to herbivory [48], suggesting that *AOS* is critical to plant defenses and implicates *AOS* in trichome regulation.

Mutations in the *AOS* gene, which encodes the essential enzyme involved in JA biosynthesis, result in a complete disruption of JA synthesis [51]. Confirming the essential role of the JA biosynthesis pathway in the initiation and morphogenesis of trichomes in cucumber, as previously reported in Arabidopsis [11], we revealed that *CsAOS* knockout lines have significantly reduced trichome density. Knockout of *AOS* mutants in Arabidopsis did not trigger trichome formation, suggesting that JA biosynthesis, catalyzed by *AOS*, is both required and sufficient for trichome induction [11]. In contrast, overexpression of *CsAOS* in cucumber led to a significant increase in trichome numbers, a phenomenon known to be mediated by JA in plants (Fig. 8A–B). The phenotypic results from the *CsAOS^OE^* lines are consistent with the importance of *CsAOS* in increasing trichome density in the overexpression lines (Fig. 8D), where spine morphology is different for the overexpression lines compared to the WT (Fig. 8D). This further suggests that JA, synthesized by *CsAOS,* is crucial for trichome formation in response to environmental cues. Other enzymes and genes, which have been shown to be involved in trichome regulation, such as those controlling trichome cell fate (*GL1* and *TTG1* in Arabidopsis), are important in regulating trichome development [52]. The *MYC2* transcription factor, which is activated by JA, increases the expression of Jasmonate ZIM-Domain (JAZ) related genes as well as other JA biosynthetic genes that feed into a positive feedback loop that amplifies JA levels and stress tolerance in tea plants [53]. Trichome development is regulated by a complex network of genetic, hormonal, and environmental signals [54, 55]. A study on Arabidopsis demonstrated that *AOS* from *Castanea crenata* enhanced resistance to *Phytophthora cinnamomi,* indicating that *AOS*-produced oxylipins, including JA, simultaneously manage defense processes and growth regulation [56]. Adapting to environmental challenges, such as herbivore attacks, which are known to trigger JA production and consequent trichome formation, requires this balance [57].

Taken together, our results strongly indicate that *CsAOS* plays a key role in the regulation of trichome development in cucumber and connects JA biosynthesis with trichome density and morphology. *CsAOS*-mediated JA signaling integrates growth and defense signals in a simple and sophisticated mechanism by which plants adapt to the environment. Although the present study establishes a regulatory link between *AOS* and trichome development, a comprehensive phenotypic dissection of *AOS* transgenic lines across tissues represents an important future direction to further refine the mechanistic framework. Furthermore, the downstream targets of *CsAOS* in trichome development and how other environmental factors affect the regulation of *CsAOS* to fine-tune plant defense responses should be explored in future studies.

## Conclusion

We showed that JA plays a major role in governing trichome development in cucumber, with the identification of JA concentration and stage of developmental growth regulating trichome density and morphology. JA controls the formation of non-GTs and GTs, and further findings suggest concentration-dependent effects, indicating the importance of JA signaling thresholds in determining trichome formation. In addition, *CsAOS* serves as a key regulator of JA biosynthesis; therefore, its identification reinforces its role in trichome initiation and morphogenesis. Our results contribute to the increased knowledge of hormonal and genetic networks. This has generated valuable insights into the process by which *CsAOS* modulates JA signaling, which will serve as a foundation for further investigations to improve plant resistance or agricultural practices through the precise regulation of trichome formation.

Future studies should investigate the genetic and transcriptional pathways that originate from *CsAOS* to promote trichome development. The current study primarily focuses on leaf trichomes; however, an important follow-up analysis will be a way to assess the impact of *AOS* transgenic lines on fruit trichome density in future projects. In addition, future research should concentrate on analyzing environmental variables such as herbivore pressure and abiotic stress, which modify both *CsAOS* expression and JA biosynthesis, to develop modern techniques for crop resilience management through agronomic practices and genetic modification. Future studies on trichome development triggered by JA must determine when JA becomes active or inactive during plant maturation for trichome formation. New information on plant strategy management between growth, defense, and reproduction may become available through this research. Extensive multi-omics methods that combine transcriptomic and metabolomic analyses would generate in-depth information regarding the genetic pathways through which JA affects plant growth control and stress response mechanisms.

## Materials and methods

### Plant materials and hormone treatments

Cucumber plants of the North China type (Chinese Long) inbred line (CCMC) and 6101–4 line were cultivated in a greenhouse at the experimental field of China Agricultural University (Beijing, China). The cultivation practices followed standard protocols. Cucumber plants were sprayed with 5 ml solutions of JA at 0.5, 1.0, and 1.5 mM JA, while the JA inhibitor was used at 1.0 mM on three to four true leaves. The control treatment was performed with 5 ml of water per plant. At three developmental stages, fruits were examined for trichomes: 5 DBF, 0 DBF, and 5 DAF. The experiment was repeated three times for each treatment.

### Phylogenetic analysis, gene selection, and sequence alignments

The complete amino acid sequence of *CsAOS* was aligned using ClustalW in MEGA11. Phylogenetic trees were reconstructed based on the maximum likelihood (ML) approach with the *p*-distance model of amino acid substitutions. Additionally, 1000 bootstrap replications upheld the validity of the resultant tree owing to the highly consistent clade stability. The MEME 5.5.7 database was used to identify motifs with complete amino acid sequences of *AOS*. The lengths of *AOS* motifs are demonstrated proportionally. *CsAOS* was selected from our RNA-seq data available in the NCBI database (accession no. PRJNA1185714). Detailed protocols of transcriptome analysis including RNA extraction, sequencing, and data processing can be found in previously published work [8].

### qRT-PCR analysis of *CsAOS* expression in different spatial and temporal contexts

We extracted total RNA from different plant tissues using a Quick RNA isolation kit (Huayueyang China), and cDNA was synthesized using a PrimeScript First Strand cDNA Synthesis Kit (TaKaRa). These tissues included stems, male and female flower buds, tendrils, and fruit at different stages. qRT–PCR was conducted with SYBR® Premix Ex Taq from TaKaRa using an Applied Biosystems 7500 real-time PCR system (Applied Biosystems). All qRT–PCRs were performed using the ubiquitin gene from cucumber as an internal control [58]. The expression data were verified using three biological replicates and three technical replicates. Gene-specific qRT-PCR primers of *CsAOS* are shown in Supplementary Table S3.

### Subcellular localization assay

The CDS of *CsAOS*, excluding the termination codon, was cloned and inserted into the pSUPER1300 plasmid between SmaI and SpeI restriction sites. The resulting plasmid was then transformed into *Agrobacterium tumefaciens* strain GV3101. An empty pSUPER1300 vector was used as control. The transformed *Agrobacterium* was resuspended in an infection buffer (10 mM MES, 20 mM AS, 10 mM MgCl_2_, pH 5.6) and used to transiently infect *N. benthamiana*. Following infection, the plants were returned to normal environmental conditions and kept in the dark for 24 h. GFP fluorescence was observed using a ZEISS LSM800 confocal microscope (Germany), with excitation/emission wavelengths of 488/510 nm (GFP) and 552/610 nm (mCherry) 72 h post-infection with the *Agrobacterium* solution. All the primers used in this study are listed in Supplementary Table S1.

### Phenotypic and scanning electron microscopy data collection

SEM was conducted on two inbred cucumber lines, CCMC and 6101–4, as well as treated and WT plants, at three distinct stages of fruit development: 5 DBF, 0 DBF, and 5 DAF. Cucumber fruit peel samples were fixed, washed, post-fixed, dehydrated, and coated using a procedure adapted from [59] and analyzed using a Hitachi S-4700 scanning electron microscope at an accelerating voltage of 2 kV [59]. Trichome density, length, and width were measured at these three developmental stages for both treated and WT plants, as well as for the mutant plants. To differentiate the treated samples from the WT under varying treatment conditions, trichome morphology, including density, size, shape, and surface features, was compared across treated and untreated samples.

In the mutant plants, accurately comparing trichome density proved challenging, as leaf expansion continues after trichome development ceases [60] and final leaf size is greatly affected by the microenvironment. Therefore, instead of comparing density, trichome numbers were assessed by examining both the adaxial and abaxial sides of the fifth true leaf under a stereomicroscope and scanning electron microscope, allowing for the determination of trichome numbers per unit leaf area in the mutant plants.

### Cucumber transformation

To perform gene editing using CRISPR/Cas9, we used the PKSE402-GFP vector. A modified dual-35S promoter was used to drive *CsAOS* overexpression vector constructed on the pCY-GFP vector backbone, and cloning was performed between BamH and SalI restriction sites. They were then introduced into *Agrobacterium tumefaciens* strain GV3101 to transform the recombinant plasmids. The transformation process was performed using the cucumber line CCMC. Cucumber seeds were sterilized under sterile conditions and then germinated on SGM (MS solid medium with 2 mg/L 6-BA). Therefore, cotyledons were excised after 40 h and infected with Agrobacterium resuspended in liquid IM medium (liquid MS medium comprising 6-BA 2 mg/L, ABA 1 mg/L, AS 200 μM, MES 1.25 mM, pH 5.2). Solid IM was used for the co-culture of infected cotyledons for 3 days. After co-cultivation, the explants were transferred to SRM (MS solid medium containing 2 mg/L 6-BA, 1 mg/L ABA, and 300 mg/L timentin) for 30 days. Fluorescence in buds was monitored by GFP fluorescence using a LUYOR-3260 GFP fluorescent flashlight (LUYOR, USA) to confirm successful transformation [61, 62].

### Analysis of endogenous JA content

We sampled the leaves of the cucumber plants. Endogenous JA content was determined using plate direct competition enzyme-linked immunosorbent assay (ELISA) after grinding the samples with liquid nitrogen—an indirect ELISA technique for the extraction, purification, and determination of endogenous JA.

### Statistical analysis

We combined phenotypic, SEM, and gene expression data to better understand the effect of JA treatment and mutations on trichome density and morphology. Statistical analysis of expression levels and phenotypic traits was conducted with Statistix 8.1 employing the two-factor LSD test and GraphPad Prism 9. We have expanded this data set to obtain a better understanding of the genetic and molecular pathways that mediate trichome development.

## Supplementary Material

Web_Material_uhag111

## Data Availability

The data supporting this article are accessible within the article itself and the online supplementary material.
