# Supplementary material for: Exploring the jasmonic acid signaling pathway: the role of CsAOS in modulating trichome density in cucumber
Source: Hortic Res. 2026 Mar 30;13(7):uhag111. doi: 10.1093/hr/uhag111 (PMC13326749; doi:10.1093/hr/uhag111)
Supplement: Web_Material_uhag111 [file web_material_uhag111.zip › Supplementary figure.docx]

**Supplementary Information**

Exploring the Jasmonic Acid Signaling Pathway: The Role of *CsAOS* in Modulating Trichome Density in Cucumber


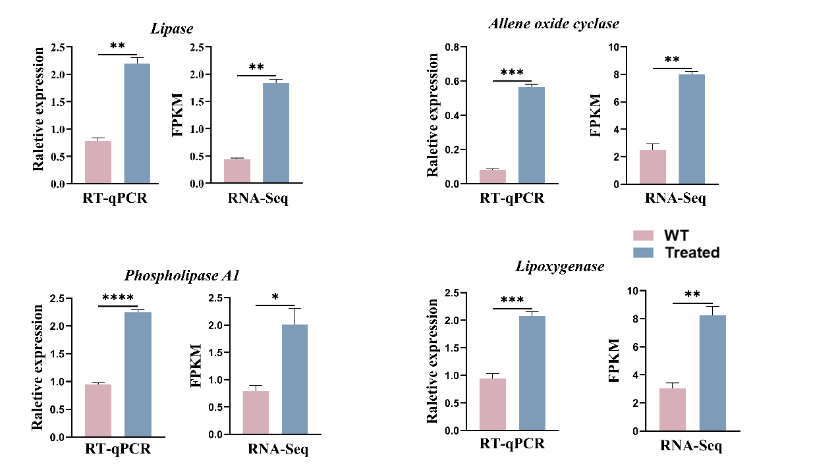


**Supplementary Figure S1:** Validation of RNA-Seq genes by real-time quantitative RT-PCR.
